# Advances, Challenges and Future Trends of Cell-Free Transcription-Translation Biosensors

**DOI:** 10.3390/bios12050318

**Published:** 2022-05-10

**Authors:** Ting Wang, Yuan Lu

**Affiliations:** Key Laboratory of Industrial Biocatalysis, Ministry of Education, Department of Chemical Engineering, Tsinghua University, Beijing 100084, China; wangting21@mails.tsinghua.edu.cn

**Keywords:** cell-free protein synthesis, cell-free biosensors, transcription-translation, performance optimization, application prospect

## Abstract

In recent years, the application of cell-free protein synthesis systems in biosensing has been developing rapidly. Cell-free synthetic biology, with its advantages of high biosafety, fast material transport, and high sensitivity, has overcome many defects of cell-based biosensors and provided an abiotic substitute for biosensors. In addition, the application of freeze-drying technology has improved the stability of such systems, making it possible to realize point-of-care application of field detection and broadening the application prospects of cell-free biosensors. However, despite these advancements, challenges such as the risk of sample interference due to the lack of physical barriers, maintenance of activity during storage, and poor robustness still need to be addressed before the full potential of cell-free biosensors can be realized on a larger scale. In this review, current strategies and research results for improving the performance of cell-free biosensors are summarized, including a comprehensive discussion of the existing challenges, future trends, and potential investments needed for improvement.

## 1. Introduction

“Biosensor” refers to the combination of bioactive materials and sensors, relying on enzymes, cells, antigens and antibodies, nucleic acids, microorganisms, and other biomass to achieve detection of a given substance [1]. The substance to be measured enters the biologically active material through diffusion, and a biological reaction occurs after molecular recognition. The information generated is then converted into quantifiable and processable electrical signals by the corresponding physical or chemical transducer and then amplified and output by the secondary instrument. In this way, the concentration of the substance to be measured can be obtained. Biosensors rely on biosensitive substances with natural selectivities, such as receptor proteins, which can achieve a very low detection limit and have the advantage of detecting relatively small amounts of target substances. With the advantages of easy construction, significant specificity, and high sensitivity, biosensors have been widely applied in varied fields, including disease diagnosis [2], environmental monitoring [3], food technology [4], etc.

**Figure 1:**
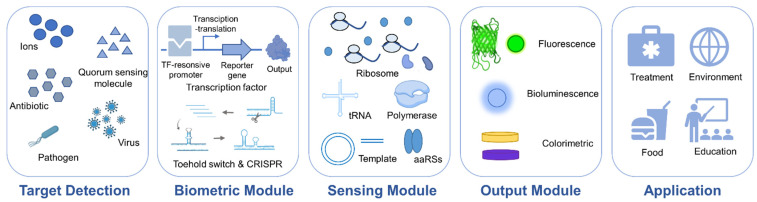
Typical systems of cell-free transcription–translation biosensors including target detection, biometric modules, sensing modules, output modules, and application scenarios.

According to the detection principle, biosensors can be divided into the following two kinds. One is based on the direct interaction or reaction between molecules, such as contact identification between an enzyme and its substrate or antigen and antibody [5,6]. Then, the sensor can capture the interaction between the sensitive material and the analyte, and turn the interaction into an identifiable signal. For example, the interaction between a field effect transistor (FET) biosensor and the target biomolecule can be directly converted into electrical signals [7,8]. The other type of biosensor is for cellular and cell-free systems based on transcription–translation processes in synthetic biology [9,10] (Figure 1).

By redesigning proteins, genes, metabolic pathways, and complex biological systems, synthetic biology can understand and transform the basic life activities of biological organisms. Thus, synthetic biology can realize rational design of genetic circuits and construct a variety of genetic devices and biological modules so as to achieve customized programming of components of each module [11]. By amplifying the initial reaction of a biological system and editing the gene regulatory system, the application of synthetic biology technology can improve the sensitivity, stability, selectivity, and analysis ability of biosensors in complex environments [12,13,14]. Therefore, the combination of synthetic biology and biosensors can realize the optimization of sensor performance and obtain new and advantageous functions in the field of sensors, which means that synthetic biology technology in the field of biosensors has shown a great potential for improvement.

Biosensing systems based on synthetic biology can be divided into cellular and cell-free sensors. Both types of biosensors have their own advantages and disadvantages. Among them, cell-based biosensors use animal cells, plant cells, or microbial cells as sensitive biometric elements; recognize and combine analytes; and use intracellular enzymes to catalyze various metabolic processes to generate signal output [15]. When a cell acts as a biological receptor, enzymes and other molecules within the cell are present in its natural environment and thus exhibit optimal activity and specificity, which has not been achieved by molecular biosensors. In addition, whole-cell biosensors can analyze and monitor drug–ligand interactions, bioactive agent interactions, environmental toxicity, etc. [16,17,18]. However, many biosensors have high requirements for a storage environment to maintain activity and a limited range of applicable analytes and rely on expensive laboratory equipment [19]. Therefore, it is necessary to develop more widely applicable and portable detection methods.

Cell-free protein synthesis refers to the process of obtaining the essential components for transcription and translation from cells and adding DNA templates in vitro to maintain the operation of gene transcription, protein translation, or metabolic processes to synthesize the target product [20]. The emergence of cell-free protein synthesis systems has promoted the development of biosensors and solved the above limitations to a certain extent [11]. Cell-free systems also provide the additional advantage of rapid response for removing barriers to transmembrane transport and avoid biosafety issues and nutritional limitations in cell storage [21]. Based on the above advantages, cell-free biosensors have attracted wide attention and achieved remarkable outcomes in the rapid detection of environmental pollutants, in clinical biomedical applications, and in the detection of disease pathogens [22,23,24].

Cell-free transcription and translation systems applied to biosensors, while bypassing many of the difficulties associated with deploying live biosensors, also present new challenges. The removal of cell membranes in the system avoids the barrier of transmembrane transport of analytes and output signal substances but also leads to the loss of a physical barrier to protect the components of the system, thus making the protein synthesis reaction more susceptible to interference from complex components in the sample to be tested [25]. In addition, freeze-drying technology can be employed to fix cell-free transcription and translation systems on paper and other substrates to improve portability [26,27] but also causes challenges such as decreased component activity and weakened sensor function [28].

In this review, we focused on cell-free transcription–translation biosensors, summarized recent achievements in the optimization of sensitivity and portability of cell-free biosensors, and discussed the challenges associated with the practical application of cell-free biosensors, as well as some of the latest trends and future prospects in the field (Table 1).

## 2. Performance Optimization of Cell-Free Biosensors

Cell-free biosensors are composed of biometric components, sensing modules, and signal output/analysis modules. When the system comes into contact with samples containing the target analyte, biometric components based on different recognition mechanisms recognize it and stimulate the response of the sensing system immediately. Then, the reporter genes downstream of the sensor system are activated and expressed, and the detection results are finally output in the form of optical signal, electrochemical signal, etc.

**Figure 2:**
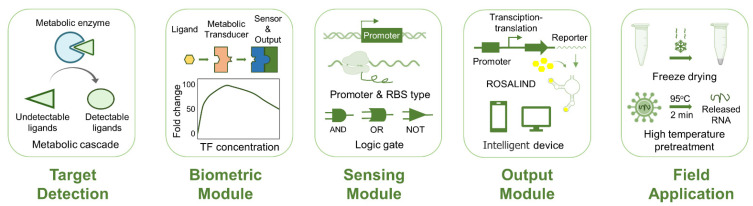
Optimization strategies in each part of cell-free transcription-translation biosensors, including target detection, biometric modules, sensing modules, output modules, and field applications.

With more extensive practical applications of cell-free biosensors, their sensitivity, response characteristics, and stability are facing higher requirements, which also promotes the continuous improvement of cell-free biosensors in practical applications. Cell-free transcription and translation systems eliminate the necessity of maintaining cell activity for gene expression, enabling the design-build-test experimental cycle to be completed more quickly and conveniently. The modules and components in the system are also given greater freedom and editable ability to optimize the performance of cell-free biosensors (Figure 2).

### 2.1. Optimization in Sensing Response

Initially, biosensors responded poorly to target molecules because of highly leaky expression, small dynamic range, and poor sensitivity, which were important issues limiting their application. In the application scenario, the detection target of the sensor mostly appears at a low concentration or only a small concentration change needs to be detected, which requires the sensor to have high sensitivity, a great signal-to-noise ratio, and dynamic range.

Optimizing the concentration of transcription factors could reduce the detection limit (LOD) of the sensor [40]. The detection limit determines whether the biosensor can detect the target analyte. In transcription–translation-based biosensor systems, such as biosensors based on the change of transcription factors to control the reported gene expression, the regulation of the concentration of transcription factors plays an important role in detecting the concentration of target proteins. Therefore, changing the concentration of transcription factors can optimize the detection limit of cell-free biosensors. Whether the optimal response requires increasing or decreasing the concentration of transcription factors in the system depends on whether the transcription factor is an activator or suppressor [41]. Voyvodic et al., reduced the detection limit of a BenR cell-free biosensor by titrating TF DNA concentrations [29]. A significant advantage of the cell-free framework is that the concentration of each component can be directly controlled by pipetting. Therefore, the optimal concentration was easily determined experimentally, which facilitated the rapid development and optimization of candidate biosensors or more complex devices that rely on them.

In addition to the concentration of transcription factors, the concentration of reporter plasmids, the type of promoter types, ribosomal binding sites (RBS), and degradation labels of the output proteins all affect the gene expression process [42,43]. They adjust the expression level of a given gene by regulating the transcriptional intensity, translation rate, and steady-state concentration of proteins, thus adjusting the dynamic range of cell-free biosensors and improving their response [22].

Some researchers also optimized the signal output/analysis module of cell-free biosensors. Lopreside et al., focused on reporter gene types to optimize cell-free biosensors. Different reporter genes have different detection efficiencies, so the selection of reporter genes is the key to sensor performance and its successful application. Lopreside et al., systematically characterized and compared a fluorescence reporter, colorimetric reporter, and bioluminescence reporter under two representative mercury and AHL-sensing biosensors, providing a new reference for reporter gene selection [44]. This work provided guidance for the rapid development of cell-free biosensors in combination with different practical application requirements, such as background signal level and manufacturing cost.

Another method of optimization is to shorten the path of signal output. Most transcription-factor-based biosensors are monitored by reporting on the transcription and translation of proteins, such as green fluorescent protein. Alam et al., developed a ligand-induced activation RNA output sensor (ROSALIND) system that combines RNA polymerase, allosteric transcription factor, and DNA template to produce fluorescent aptamers for ligand detection. The fluorescent adaptor was used to bypass the translation step, thus improving the response speed of the sensor and shortening the response time [45].

In addition, some of the synthetic biology tools that have been successfully used in cell-based biosensor systems could also be used to improve the performance of cell-free biosensors and extend their applications. Wang et al., introduced a set of modular and gain-tunable genetic amplifiers into sensors, enhancing the output dynamic range by amplifying the transcription signal by changing the expression level of the underlying ligandless activated protein in the system [46,47]. Voyvodic et al., combined metabolic engineering strategies with cell-free biosensing systems by adding specific enzymes to biosensor modules. Metabolic enzymes converted molecules with no known corresponding transcription factors into metabolites that could trigger transcriptional reactions. The metabolic cascade solved the problem of limited transcription factors and effectively expanded the range of chemicals that can be detected by cell-free biosensors [29]. Bonnet et al., introduced logic gates into genetic circuits to realize the regulation and integration of multiple input signals [48]. Ma et al., performed two-input and three-input OR and logic gates using an integrated RNA system and sequent-independent input RNAs to achieve accurate recognition of severe acute respiratory syndrome coronavirus 2 in saliva samples [49]. Hunt et al., used mathematical simulations of enzyme kinetics to optimize the biosensor assay, ultimately lengthening its readable window by fivefold and improving sensor signal strength by twofold, providing insight for engineering rapid and field-deployable CFPS biosensors [31]. Thakur et al., showed that nanoparticle scaffolding of the CFPS crosslinks the QDs into nanoaggregate structures while enhancing the production of functional recombinant super-folder green fluorescent protein and phosphotriesterase, an organophosphate hydrolase. This enhancement has the potential to improve CFPS in general and specifically CFPS-based biosensors (faster response time) [50].

### 2.2. Optimization of Portability and Stability

As the prospect of cell-free biosensors becomes broader in practical application, the stability and portability of cell-free biosensors are facing higher requirements. Each module and component in the sensor needs to maintain its activity and function during storage and, preferably, also maintain stability in a fluctuating environment to ensure the reliability of the target substance detection results. In addition, biosensors need to be more portable for field deployment and application.

Cell-free biosensors mainly rely on two kinds of cell-free transcription and translation systems, namely extract systems and reconstructive in vitro systems (protein synthesis using recombinant elements, PURE). In the laboratory, the components of the cell-free system need to be refrigerated or frozen. The requirements of cold-chain storage are associated with difficulties in the practical application of biosensors. On the one hand, they are not suitable for long-distance transportation, and on the other hand, they are difficult to apply in countries and regions with relatively scarce resources and difficult-to-achieve temperature control.

In order to improve the storage stability of cell-free systems, Pardee et al., first applied freeze-drying technology in the preservation mode of toehold switch biosensors, providing an effective method for cell-free systems to be stored at relatively high temperatures [51]. Salehi et al., compared the storage stability of freeze-dried and normal extracts against the cell-free expression system of cytotoxic protein onconase. They demonstrated that lyophilized extracts maintained their function for longer at high temperatures [28]. Hunt et al., employed lyophilized cell-free protein synthesis and toehold switch riboregulators to develop a promising paper-based nucleic acid diagnostic platform activated simply by the addition of saliva [52]. Smith et al., demonstrated that freeze drying provided the additional benefit of killing residual bacterial contamination in the system, which was favorable for the stability of the system [53]. Freeze-drying systems have been shown to maintain their function for three months to a year [28,54].

Freeze-drying technology can create sterile and abiotic materials with fundamental transcription and translation properties. Therefore, paper-based biosensors could be stored and distributed readily and could be easily activated by adding water. The improved stability and the reduced storage space requirements of cell-free systems have created a path for moving biosensors out of the lab and into the field and closer to application in situ. Cell-free systems could be freeze-dried on supports, such as microtubes, microporous plates, and inexpensive paper [33]. These materials could be used as a high-capillary substrate to accommodate small volumes of molecules and biochemical reactions and greatly reduce the cost of production, transportation, and storage. The average cost of transcription–translation is only a few pence per microliter, creating further conditions for the practical application of cell-free biosensors [55].

In addition to the requirements for storage conditions, cell-free biosensors rely on expensive and specialized equipment, which also limits their practical application. Verosloff et al., developed a cell-free biosensor based on isothermal amplification technology, which achieved nucleic acid amplification of target plant pathogens only by hand heating, eliminating the need for a PCR apparatus [56]. Alexander et al., developed a dual-filter system for detecting sfGFP fluorescence by combining the camera and flash of a smartphone with a filter, eliminating the need for specialized equipment to detect fluorescence intensity, such as a microplate reader [26]. These measures minimized the equipment required for field use and provided a more convenient method for cell-free biosensors to be used in field investigations.

## 3. Challenges and Future Trends

Cell-free protein synthesis system (CFPSs) perform the transcription and translation process in a completely open environment and coassemble with a DNA template to form a cell-free biosensor. With their unique advantages of “bottom-up” research on the origin of life and rapid development and design of biological circuits, cell-free biosensors have gained extensive attention and rapid development in recent years, and the sensitivity, output dynamic range, response speed, stability, and portability of such systems have all been improved. Cell-free biosensors have shown significant advantages in environmental detection, medical diagnosis, and many other fields. However, there is still a lag in large-scale commercial applications of cell-free biosensors, and many challenges need to be addressed before they can be widely applied for global monitoring and independent use by the public.

### 3.1. Risk of Sample Interference

A completely open cell-free environment not only brings the advantages of strong chemical tolerance and short response time to the system but also the defect of being easily interfered with by other substances in the sample. Conversely, whole-cell biosensors have membranes that act as a natural barrier, protecting them from interference by other substances in the environment. Ma et al., developed a whole-cell biosensor based on *Escherichia coli* for tetracycline detection. According to analysis of actual water samples, the detection data of sewage samples collected from fish ponds were almost the same as the data of laboratory standard water samples, indicating that the influence of sample matrix on the sensitivity of the whole-cell biosensor of tetracycline was negligible [57]. Guo et al., developed a detection strip platform based on a whole-cell microbial biosensor. The biosensor was used for the qualitative detection of soluble and insoluble mercury contamination in cosmetics without prior treatment. An instrument-independent method for on-site detection of mercury contamination in cosmetics was developed [58]. It can be seen that whole-cell biosensors would not be easily interfered with in the sample matrix.

**Figure 3:**
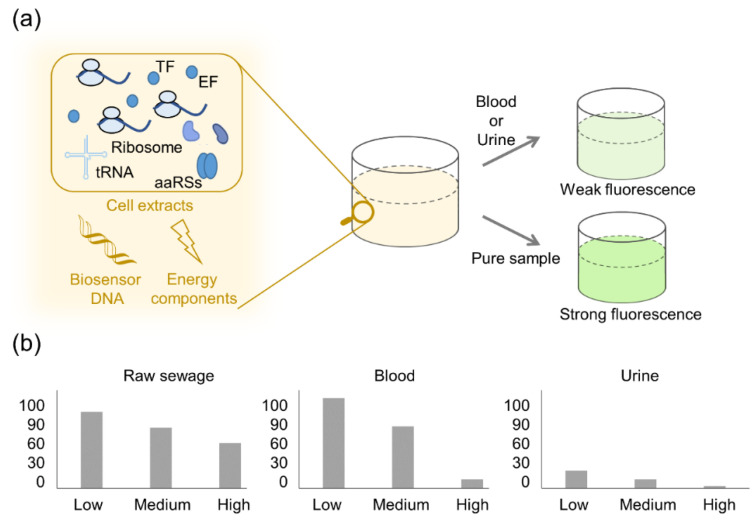
Protein production capability of CFPS in the presence of actual and pure samples. (**a**) Compared to the pure sample prepared in the laboratory, model protein GFP is expressed in actual samples, such as human blood and urine. TF represents transcription factor, EF represents elongation factor, and aaRSs represents aminoacyl tRNA synthetases. (**b**) The fluorescence signal changes with various kinds and concentrations of detecting samples.

However, the absence of a cell membrane in a cell-free biosensor results in the loss of a natural physical barrier in the system compared to a cell-based biosensor. However, natural environmental samples or medical clinical samples, such as sewage and urine, are mostly mixtures of complex components. Non-target substances in the samples may interfere with sensor components or chelate with the substances to be detected, resulting in false-positive or false-negative results (Figure 3). Salehi et al., examined the performance of a cell-free transcription-translation system in various samples of untreated water, raw sewage, and human body fluids. The results showed that CFPS activity decreased in all samples compared to high-purity laboratory samples. The activity of CFPS decreased significantly in human urine, which might be due to the high concentration of protein denaturing urea (about 280 mM) in human urine [38]. Therefore, one of the key directions of future trends of cell-free biosensors is to overcome the potential inhibition effects and improve their robustness in complex samples.

**Figure 4:**
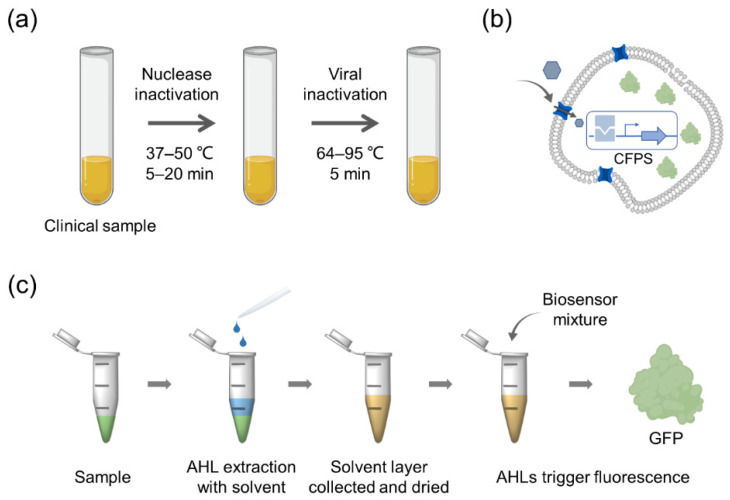
Series of methods to reduce the sample interference of cell-free biosensors. (**a**) Heating unextracted diagnostic samples to obliterate nucleases. (**b**) Artificial cells incorporate cell-free biosensing strategies and synthetic membrane components. (**c**) Workflow of solvent extraction to concentrate AHLs from sputum, followed by concurrent analysis by cell-free biosensors.

To date, some cell-free biosensors have been successfully applied to detect target substances in complex samples (Figure 4). Among them, an effective way to reduce sample interference is to add pretreatment steps to the sample to minimize the influence of non-target substances. Before using a cell-free biosensor to detect the quorum-sensing molecule 3-OXO-C12-HSL in human sputum samples, Wen et al., mixed the sample with an organic solvent (ethyl acetate), separated and collected the organic phase, and dried and concentrated the AHLs in the sample. The cell-free biosensor for quorum sensing molecule detection quantitatively measured quorum-sensing molecules at the nanoscale comparably to liquid chromatography [59]. Salehi et al., used cell-free biosensors to detect endocrine disruptors in human blood and urine samples [25]. They added RNAse inhibitors to the samples to overcome adverse sample matrix effects and significantly enhanced reporter protein synthesis in cell-free systems. Soltan et al., used CFPS systems to produce RNAse inhibitors to reduce the cost associated with using commercial reagents [60]. Myhrvold et al., developed the HUDSON (heating unextracted diagnostic samples to obliterate nucleases) method to preprocess samples, realizing direct detection of viruses from body fluids [61]. Zhang et al., demonstrated that Escherichia coli lysate-based cell-free biosensors coupled with a personal glucose monitor (PGM) can enable on-site analyte quantification, and this lysate metabolism allowed for one-pot removal of glucose present in complex samples (such as human serum) without confounding target quantification [62].

The combination of CFPS systems with synthetic materials, such as artificial cells, is also a promising way to develop novel biosensors [63]. Recent advances in the development of artificial cells have shown that cell-free biosensors can be encapsulated in bilayer membranes to detect a variety of analytes and environmental changes, expanding the capabilities of cell-free sensing of forces and light and expanding the operating range of cell-free biosensors [64]. At the same time, the chassis in the system could also act as an additional “gate” to regulate the transmission of environmental information. Similar to the biological barrier of cell membranes, synthetic material encapsulation could reduce environmental interference, protecting cell-free components and playing a positive role in cell-free biosensing [64,65]. Whereas membrane transport of the artificial cells that encapsulate CFPS systems remains an unsolved barrier, synthetic materials embedded within cell-free biosensor systems for artificial cells have great potential for future application in real scenarios.

There are also cell-free biosensors that have been tested for detection in untreated samples. Salehi et al., developed cell-free biosensors to detect EDCs that maintained high protein production levels in a variety of untreated water samples (faucets, ponds, snow, and storms) and water samples from all stages of the wastewater treatment plant, which showed outstanding robustness [38]. Thavarajah et al., developed cell-free biosensors that could detect fluoride in real water sources in the field [54]. These studies preliminarily demonstrated the possibility of cell-free biosensors in the application of complex samples. However, considering the diversity of potential interference substances in mixture samples and genetic circuits in varied biosensors, whether cell-free biosensors are effective in complex samples still needs further testing, as well as mechanism exploration and interpretation. Only in this way can we provide theoretical support for the artificial design and assembly of more robust cell-free biosensors.

### 3.2. Standards and Activity Maintenance

Most cell-free biosensors rely on cellular extract systems for transcription and translation, including template DNA, cellular crude extract, 19 amino acids, various inorganic ions, etc. [66]. Due to the influence of many factors during the preparation of such systems, such as the difference of operators, the difference of extract quality, enzyme activity, etc., there will be differences between batches in cell-free systems, which will affect the stability and standards of the biosensors. In addition, the source strain of cell extracts also affects the standard of CFPS biosensors. The most common cell extracts come from *E. coli*, whereas Fabrega et al., designed a biosensor using *C. acnes* CFS to assay a pNA14 plasmid containing green fluorescent protein (FMN-GFP) gene reporter controlled by a modulable promoter [67].

The difference between batches of extract systems can be reduced as much as possible by mass preparation, automatic preparation, and full mixing of components [68]. In addition, the PURE system (protein synthesis using recombinant elements) purifies and assembles the individual components necessary for gene expression into the reaction, which has higher repeatability and stability. To date, there has been strict quality control of the market PURE system. However, the preparation of the PURE system involves the purification of a large number of components, which means complex steps and high cost, so the price of commercial PURE systems is still relatively expensive. As a result, common cell-free biosensors still mainly rely on the extract system. Therefore, the future trend of cell-free biosensors in improving quality control standards is to standardize and automate the preparation of the extract system, whereas preparation of PURE systems should improve efficiency and reduce production costs.

In terms of system stability, although freeze-drying technology allows cell-free systems to be preserved at room temperature, freeze-drying platforms still have some disadvantages. For example, cell-free biosensors require an inert gas and silica dry-pack environment to prevent oxidative damage and hydrolytic damage in order to maintain their function for about a year. Moreover, most cell-free biosensors rely on cell-free protein synthesis systems based on T7 RNA polymerase. Although the system can maintain its original detection function after freeze drying, the activity of the system reduces in the process, and the output signal decreases with the increase in storage time [28]. Cell-free biosensors that rely on activation or inhibition of polymerases from Protoescherichia Coli are currently still unable to maintain their function after freeze drying.

In view of the above shortcomings of the freeze-drying system, many scholars have proposed other solutions to improve the stability of cell-free biosensors. Karig et al., used saccharol trehalose to protect cell-free components, enabling them to be exposed to high temperatures of 37 °C and atmospheric conditions for several months after high-temperature drying in an oven, maintaining sensor function while killing *Pseudomonas aeruginosa* [69]. Attempts have also been made to separate cell-free components into hydrogels [70], protein-based structures [71], and polymer substrates [72] to maintain the stability of sensor systems. To comprehensively optimize the activity and preservation stability of cell-free biosensors, this is still one of the important trends in future development.

## 4. Conclusions

Cell-free protein synthesis systems rely on their unique advantages to provide a brand new and competitive platform for biosensors. CFPS has the advantages of high sensitivity, short response time, and high editability due to its openness and rapid prototyping. Cell-free systems open up space for the construction, development, and design of biosensors, which leads to a very wide application prospect for cell-free biosensors. Many scholars have been committed to further improving the performance of cell-free biosensors. The selection and concentration optimization of components in the system, the modification of genetic circuits, and the integration of CFPS with other technologies, such as microfluidic control and mathematical modeling, have continuously optimized the response characteristics of cell-free biosensors. In practical applications, freeze-drying systems based on portable substrates have also enhanced the stability and portability of cell-free biosensors.

Cell-free biosensors have the potential to change the quality and efficiency of human life. For example, flexible, biocompatible, wearable devices for the monitoring of vital signs and detection of heavy metals and pesticide residues in agricultural land are of great significance to food safety and human health. However, there are still many challenges that need to be addressed before cell-free biosensors can be commercialized. The detection effect of sensors in complex samples in real scenarios, the control of sensor activity and quality standards, and the sensing performance of cell-free biosensors are all directions for further optimization in the future.

In addition, in order to set up an integrated platform that can easily achieve field deployment, further work is needed to enhance the multiplexing performance, specificity, and sensitivity of cell-free biosensors. For example, Martinez et al., developed a paper analysis based on microfluidic devices (μPADS) that can be used for integration with cell-free systems [73]. Suvanasuthi et al., demonstrated a simple technique for using common polylactic acid (PLA) filament and wax filament to create hydrophobic barriers on paper for μPADs using a commercialized 3D printer [74]. As our understanding of genetic circuitry deepens, the design of genetic circuitry in cell-free biosensors will become increasingly complicated. Researchers can develop new sensing mechanisms to extend the detection range of biosensors and improve the specific detection of target molecules to limit interference in the samples. In addition, most of sensors described in this paper have been used for the detection of chemical inputs; efforts to expand the application scope of cell-free biosensors can also start from non-chemical stimuli, such as the exploration of gravity sensors conducted by Chen et al., to broaden the application scope [75]. Finally, the commercialization of cell-free biosensing platforms requires the establishment of more regulatory standards and approval processes, and the safety and effectiveness of products need to be accurately verified. In recent years, teaching packages concerning cell-free biosensors were developed for education in high school, guiding students to use cell-free technology to identify fruit varieties, learn antibiotic mechanisms, etc. [39,76]. Cell-free technology has been transferred to the next generation in the classroom, and a large number of new forces will continue to participate in research in the field of cell-free biosensors in the future, providing broad prospects for development.

In this review, we focused on cell-free biosensors based on transcription-translation systems, but there are still many other classes of cell-free biosensors that were been included. As the field continues to evolve, the combination of anti-interference encapsulation technology, more complex genetic circuitry, and improved sensor stability in metabolically active states could allow cell-free biosensing platforms to be deployed in environments outside the laboratory. With the integration of cell-free systems with other materials and technologies, more types and functions of cell-free biosensors will be developed in the future, building interconnected platforms and enabling them to be used in more fields, both practically and commercially.

## Figures and Tables

**Table 1:** Summary of the performance of existing cell-free transcription-translation biosensors.

Target Substance	Limit of Detection or Detection Range	Response Time	Output	Advantages	Application	Reference
Benzoic Acid	10 μM	~1 h	sfGFP	Expands the range of molecules detectable by combining synthetic metabolic cascades with transcription-factor-based networks.	Disease Diagnosis	[29]
12 Amino Acids: Ala, Cys, Gly, His, Pro, Ser, Thr, Trp, Asp, Asn, Glu, and Gln	0.1–1 μM	1 h	sfGFP	No need for chemical treatment or chromatographic separation steps, offering a rapid and economical alternative.		[30]
3OC_12_HSL	0.5 μM	3 h	LacZ, XylE	Embedded in paper by freeze-drying, stable at room temperature, and activated by simply adding water.		[3]
hERβ, Human Estrogen Receptor β	30 nM	A few minutes	β-lac	Using mathematical simulations of enzyme kinetics to optimize the biosensor assay.		[31]
hTRβ, Human Thyroid Receptor	3 nM					
Theophylline	1 mM	<90 min	lacZ	Wearable devices; comparable detection limits to those of current laboratory methods.		[32]
Ebola RNA	300 nM					
Zika Virus	2 aM	2.5 h	lacZ	Portable paper-based detection using NASBA to avoid the use of a PCR apparatus.		[33]
Chikungunya Virus	5 fM					
Mercury	6 μg/L	~1 h	sfGFP	Developing a two-filter system in combination with a conventional smartphone without the need for expensive hardware.	Food Technology	[26]
Vanillin	1 mM	75 min	deGFP	A combination of the generation of variants coupled with in vitro screening, serving as a framework for designing new sensors for other target compounds.		[34]
Tetracycline	10–10,000 ng/mL	<90 min	firefly luciferase (LucFF)	A wider detection range is achieved by eliminating toxic effects, increased sensitivity as a result of better optimization possibilities, faster assays with minimal preparation times, and a GMO-free alternative to whole-cell sensors.		[35]
Arsenic	0.5 μM	2 h	XylE	Embedded in paper by freeze drying, stable at room temperature, and activated by simply adding water.	Environmental Monitoring	[3]
Zinc	2.5 μM	Few minutes	Binding of DFHBI-1T by 3WJdB activates its fluorescence	Using ROSALIND to reduce interference and improve sensitivity; stable at room temperature for at least 2.5 months and retain function.		[36]
Copper	5 μM					
Lead	1.25 μM					
Uric Acid	50 μM					
Doxycycline	1.25 μM					
SCB1, a Streptomyces coelicolor QS molecule	0.125–2.5 nM	30 min	sfGFP	Appling cell-free E. coli protein synthesis to screen QS molecules of streptomyces for the first time.		[37]
Endocrine Disrupting Molecules (EDC)	9 nM	30 min	β-lac	A simple, colorimetric readout facilitates field deployment.		[38]
Isoamylol	25 mM	20 h	Alcohol Acetyltransferase (ATF1)	An inexpensive, easy-to-use synthetic biology education kit.	Teaching Activities	[39]
Specific DNA Templates	/	20 h	eforRED, dTomato, mOrange, sfGFP, Aquamarine			
